# A rare isolated cutaneous metastatic mass after colon cancer resection

**DOI:** 10.1093/jscr/rjab571

**Published:** 2021-12-30

**Authors:** Suysen Hung Fong, Neethi Narasimha, Rishabh Thakkar, Subhasis Misra, Darshan Thakkar

**Affiliations:** Brandon Regional Hospital, HCA Healthcare/USF Morsani College of Medicine GME, Brandon, FL 33511, USA; Brandon Regional Hospital, HCA Healthcare/USF Morsani College of Medicine GME, Brandon, FL 33511, USA; Brandon Regional Hospital, HCA Healthcare/USF Morsani College of Medicine GME, Brandon, FL 33511, USA; Brandon Regional Hospital, HCA Healthcare/USF Morsani College of Medicine GME, Brandon, FL 33511, USA; Brandon Regional Hospital, HCA Healthcare/USF Morsani College of Medicine GME, Brandon, FL 33511, USA

## Abstract

Metastatic cutaneous lesions from colorectal in origin are extremely rare, and especially without any visceral metastasis. Due to its poor response to chemotherapy, it is a poor prognostic indicator with a 1–6 month(s) death rate. Routine screening colonoscopy should be highly encouraged. This case is about a patient with obstructing, bleeding right colon mass and metastatic cutaneous soft tissue mass postcolonic mass resection. The biology and the mechanism of these metastatic lesions are not well understood, and they can be mistaken with any other primary soft tissue malignancy.

## INTRODUCTION

Colorectal cancer is the third most common malignancy in the USA and the second deadliest malignancy in both sexes [1]. Colon cancer may present sporadically and 70% of the time usually presents in people older than 50, which tends to be associated with environmental factors. It may also present through familial clustering (20%) and inherited syndromes (10%) [1]. Both the incidence and death rate of colon cancer has decreased due to the increase in cancer screenings and better therapy modalities. However, the most important prognostic indicator is the pathological staging at presentation [1, 2]. Advanced staging such as metastasis carries a poor prognosis with an overall survival rate of 5% at 5 years [1]. Most common sites of metastasis involve the liver, lung, visceral lymph nodes and peritoneum. Meanwhile, isolated cutaneous metastatic disease from colorectal cancer is rare.

## CASE PRESENTATION

**Figure 1:**
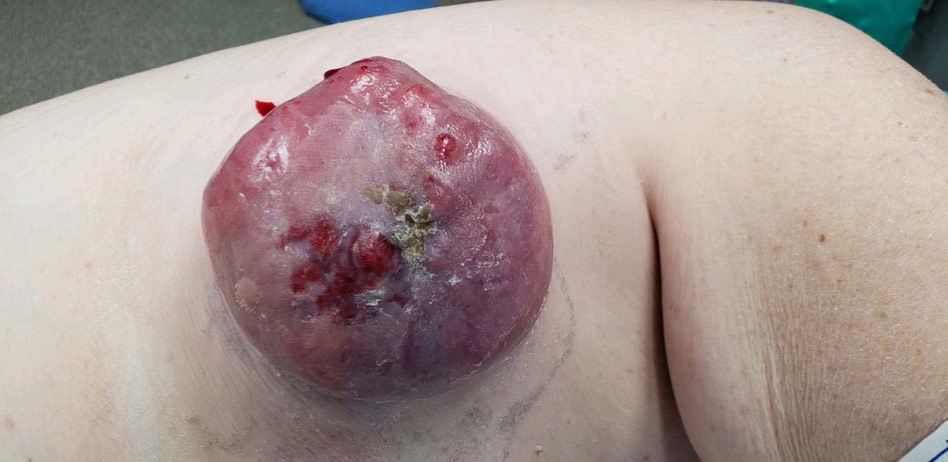
Right chest wall cutaneous mass.

**Figure 2:**
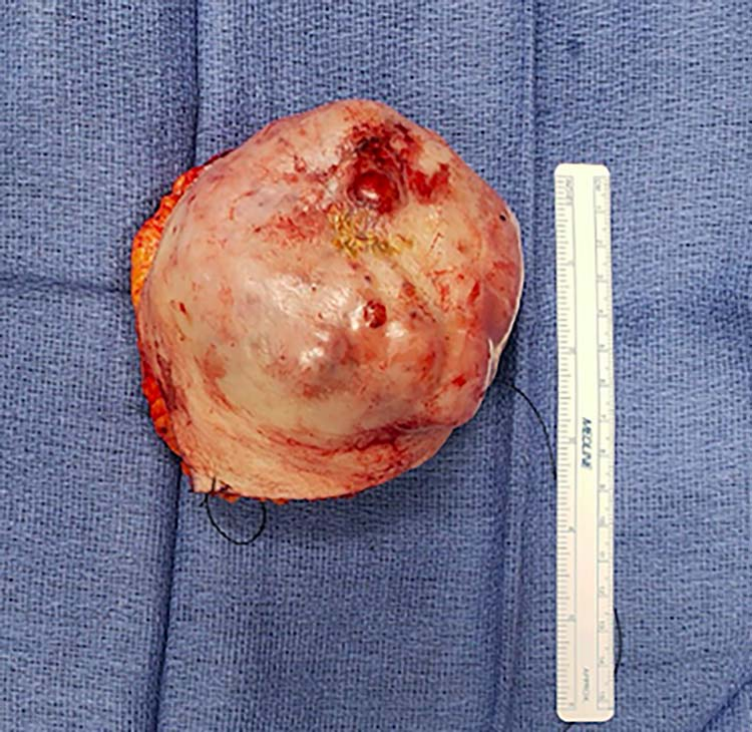
Gross specimen of right chest wall cutaneous mass.

A 70-year-old female presents with weakness and syncope without any prior history of a screening colonoscopy but has a history of gastric ulcer. Her hemoglobin was 7.2 on admission. She had a positive occult blood in stool. She then underwent an EGD and colonoscopy and was found to have a necrotic obstructing mass at the hepatic flexure. CT scan of abdomen and pelvis showed no evidence of any liver lesion or enlarged visceral lymph nodes. It was also noted that she had a large mass on the right chest wall with a central ulcer. She underwent a laparoscopic extended right hemi-colectomy with ileo-colic anastomosis. The pathology was evident of a metastatic invasive adenocarcinoma of the colon that was invading into the muscularis propria and the pericolonic tissue. One out of twenty-four lymph nodes tested positive and molecular tests as BRAF with microsatellite unstable. Post-testing, the patient was subsequently staged, T3N3Mx. During her postoperative visit, an evaluation of the upper right chest wall mass was discussed as it had been present for 2 years and was now causing the patient discomfort (Fig. 1). On MRI, her mass was 7.2 × 8-cm multiloculated along the latissimus dorsi. She underwent a total excision of the mass (Fig. 2), and her pathology was consistent with metastatic adenocarcinoma of colorectal origin. Subsequently, she was scheduled to undergo chemotherapy.

## DISCUSSION

Metastasis of colon cancer upon presentation is a very poor prognostic indicator that accounts for more than 10% of newly diagnosed colon cancer cases at the time of presentation [3]. The most common sites of metastasis are the liver, lung, visceral lymph nodes and peritoneum [1, 2, 3]. Meanwhile, cutaneous metastasis secondary to internal malignant neoplasm is very rare, ranging from 0.7 to 0.9% of all malignancies [3]. They may also appear two years after the detection and resection of the primary tumor [6, 7, 8]. Cutaneous metastasis from colorectal cancer occurs in about 3–6% of the cases, which most commonly present in the abdomen followed by the extremities, perineum, head, neck and penis [3, 4]. The lesions manifest as painless, rapidly growing subcutaneous masses with an intact epidermis [4]. Other macroscopic features may involve nodular lesions, vegetant and cystic appearances, with or without ulceration [2]. Its gross and macroscopic appearance has mimicked squamous cell carcinoma in some case reports (6.)

The mechanism of the cutaneous metastasis is not well understood. However, given that these metastatic lesions present close to the primary cancer, dissemination via direct extension of the primary tumor to the overlying skin is implicated [3]. This is supported by the most common site of metastasis being the abdomen, especially around the surgical scars where the primary malignancy was removed [2]. Other methods of metastasis may arise from lymphogenous spread, intravascular dissemination and spread along the embryonal remnant such as the urachus [4]. Cutaneous metastasis often presents simultaneously with liver, lung or peritoneal metastasis; however, there are some cases when skin metastasis may present as a single isolated lesion, which is quite rare [6, 7].

The diagnosis of cutaneous metastasis is based on the morphologic appearance, histomorphology and immunohistochemistry of the lesions [5, 6]. The most common sites for cutaneous colorectal metastasis have been reported as follows: rectum (55%), sigmoid colon (17%), transverse colon (9%), rectosigmoid (7%), cecum (4%) and ascending colon (4%) [7]. In a recent retrospective study done by Parente et al. (*n* = 29), 31% of the cutaneous metastases were present from the left colon, 45% from the right colon, 17% from the rectum and 8% from the transverse colon and anus.

The prognosis is very poor with average survival rate being at 18 months, ranging from about 1 to 34 months [7, 8]. Once diagnosed, more than two-thirds of the patients will die within the first 6 months [3]. Previous studies have shown that patients with a BRAF mutation have a poor prognostic factor in colorectal cancer; however, the optimal treatment for skin metastases with BRAF mutations needs to be further explored. For isolated lesions, wide local excision is the preferred treatment for cutaneous metastatic disease along with chemotherapy, radiotherapy and targeted therapy [5]. Other studies propose that a typical resection with 1-cm margins with normal skin is recommended in order to assess lymphovascular invasion by the pathologist [4, 6]. Due to its dismal prognosis, some debate that reconstruction is problematic as patients may require palliative radiotherapy and subsequent palliative chemotherapy [8, 9].

Cutaneous metastasis from colorectal cancer is a rare phenomenon and its presence is a poor prognostic indicator. The median survival rate is approximately 6 months and treatment options only include wide local excision with palliative therapy. For more effective treatments, further exploration is required, especially for patients presenting with BRAF mutation.
